# Light curves and colours of the ejecta from Dimorphos after the DART impact

**DOI:** 10.1038/s41586-023-05852-9

**Published:** 2023-03-01

**Authors:** Ariel Graykowski, Ryan A. Lambert, Franck Marchis, Dorian Cazeneuve, Paul A. Dalba, Thomas M. Esposito, Daniel O’Conner Peluso, Lauren A. Sgro, Guillaume Blaclard, Antonin Borot, Arnaud Malvache, Laurent Marfisi, Tyler M. Powell, Patrice Huet, Matthieu Limagne, Bruno Payet, Colin Clarke, Susan Murabana, Daniel Chu Owen, Ronald Wasilwa, Keiichi Fukui, Tateki Goto, Bruno Guillet, Patrick Huth, Satoshi Ishiyama, Ryuichi Kukita, Mike Mitchell, Michael Primm, Justus Randolph, Darren A. Rivett, Matthew Ryno, Masao Shimizu, Jean-Pierre Toullec, Stefan Will, Wai-Chun Yue, Michael Camilleri, Kathy Graykowski, Ron Janetzke, Des Janke, Scott Kardel, Margaret Loose, John W. Pickering, Barton A. Smith, Ian M. Transom

**Affiliations:** 1 SETI Institute, Carl Sagan Center, Mountain View, CA USA; 2 Unistellar, Marseille, France; 3 Department of Astronomy and Astrophysics, University of California, Santa Cruz, CA USA; 4 Heising-Simons 51 Pegasi b Postdoctoral Fellow, New York City, NY USA; 5 Astronomy Department, University of California, Berkeley, CA USA; 6 Centre for Astrophysics, University of Southern Queensland, Toowoomba, Queensland Australia; 7 Department of Earth, Planetary, and Space Sciences, University of California, Los Angeles, CA USA; 8 Unistellar Citizen Scientist, Le Tampon, France; 9 Unistellar Citizen Scientist, Saint-Paul, Réunion; 10 Unistellar Citizen Scientist, La Rivière, Réunion; 11 Armagh Observatory and Planetarium, College Hill, Armagh, UK; 12 The Travelling Telescope, Nairobi Planetarium, Nairobi, Kenya; 13 Unistellar Citizen Scientist, College Hill, UK; 14 Unistellar Citizen Scientist, Nairobi, Kenya; 15 Unistellar Citizen Scientist, Tsuchiura, Japan; 16 Unistellar Citizen Scientist, Osaka, Japan; 17 Unistellar Citizen Scientist, Caen, France; 18 Community College of Allegheny County, Pittsburgh, PA USA; 19 Unistellar Citizen Scientist, Schenley, PA USA; 20 Unistellar Citizen Scientist, Chigasaki, Japan; 21 Unistellar Citizen Scientist, Kajiki Aira, Japan; 22 Unistellar Citizen Scientist, Oklahoma City, OK USA; 23 Unistellar Citizen Scientist, Austin, TX USA; 24 Unistellar Citizen Scientist, Athens, GA USA; 25 Unistellar Citizen Scientist, Lake Macquarie, New South Wales Australia; 26 Unistellar Citizen Scientist, Milwaukee, WI USA; 27 Unistellar Citizen Scientist, Shimoishii, Japan; 28 Unistellar Citizen Scientist, Saint-Gilles, Réunion; 29 Unistellar Citizen Scientist, Raleigh, NC USA; 30 Unistellar Citizen Scientist, Hong Kong, China; 31 Unistellar Citizen Scientist, Auckland, New Zealand; 32 Unistellar Citizen Scientist, San Francisco, CA USA; 33 Unistellar Citizen Scientist, San Antonio, TX USA; 34 Unistellar Citizen Scientist, Toowoomba, Queensland Australia; 35 Palomar Community College, San Marcos, CA USA; 36 Unistellar Citizen Scientist, San Marcos, CA USA; 37 Unistellar Citizen Scientist, San Diego, CA USA; 38 Department of Medicine, University of Otago, Christchurch, New Zealand; 39 Emergency Care Foundation, Christchurch Hospital, Christchurch, New Zealand; 40 Unistellar Citizen Scientist, Christchurch, New Zealand; 41 Unistellar Citizen Scientist, Campbell, CA USA; 42 Hamilton Astronomical Society Observatory, Hamilton, New Zealand; 43 Unistellar Citizen Scientist, Cambridge, New Zealand

**Keywords:** Asteroids, comets and Kuiper belt, Astronomical instrumentation, Research management

## Abstract

On 26 September 2022, the Double Asteroid Redirection Test (DART) spacecraft struck Dimorphos, a satellite of the asteroid 65803 Didymos^1^. Because it is a binary system, it is possible to determine how much the orbit of the satellite changed, as part of a test of what is necessary to deflect an asteroid that might threaten Earth with an impact. In nominal cases, pre-impact predictions of the orbital period reduction ranged from roughly 8.8 to ﻿17 min (refs. ^2,3^). Here we report optical observations of Dimorphos before, during and after the impact, from a network of citizen scientists’ telescopes across the world. We find a maximum brightening of 2.29 ± 0.14 mag on impact. Didymos fades back to its pre-impact brightness over the course of 23.7 ± 0.7 days. We estimate lower limits on the mass contained in the ejecta, which was 0.3–0.5% Dimorphos’s mass depending on the dust size. We also observe a reddening of the ejecta on impact.

## Abstract (web summary)

Optical observations of Dimorphos, a satellite of the asteroid 65803 Didymos, before, during and after the impact of the DART spacecraft, from a network of citizen science telescopes across the world are reported.

## Main

**Fig. 1:**
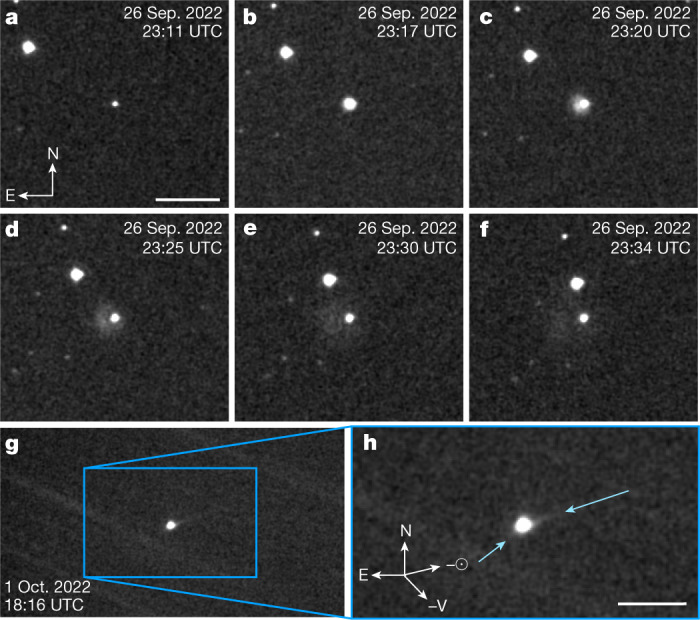
eVscope observations of the impact, ejecta and tail. **a**–**f**, The initial ejecta plume from DART’s impact into Dimorphos as observed from L’Étang-Salé, Réunion. **a**, Didymos before the DART impact. Scale bar, 100′′. **b**–**f**, Didymos after the DART impact, where **b** is roughly 6 min, **c** is 9 min, **d** is 14 min, **e** is 19 min, and **f** is 23 min after panel **a**. Each panel **a–f** is comprised of a stack of 11 4-s exposures. The compass and image scale in **a** applies to **b**–**f** as well. The fast-moving ejecta plume moves eastwards on the plane of the sky and dissipates over time, from **a**–**f**. **g**,**h**, Two tails (solar and antisolar directions) developed from the ejecta produced by the DART spacecraft roughly 113.7 h after impact into Dimorphos. The image in **h** is a zoomed-in version of the image in **g**. This image is a median stack of 1,205 4-s exposures as observed from Nagahama, Japan. The two light-blue arrows mark the two tails visible to an eVscope as visual aids. Scale bar, 70′′.

Four Unistellar eVscopes captured observations of Didymos during the Double Asteroid Redirection Test (DART) impact into Dimorphos on the night of 26 September 2022. Of all telescopes that observed the DART impact, from the ground and space, the eVscopes were among the smallest with apertures of 112 mm. Three eVscopes were located on Réunion Island and one in Nairobi, Kenya. Figure 1 shows eVscope images taken before and after the impact as well as the ejecta produced by the impact. We observe a fast-moving ejecta plume moving eastwards on the plane of the sky and spreading over a timescale of minutes, as well as slower-moving ejecta that morphologically appear to have formed a coma.

**Fig. 2:**
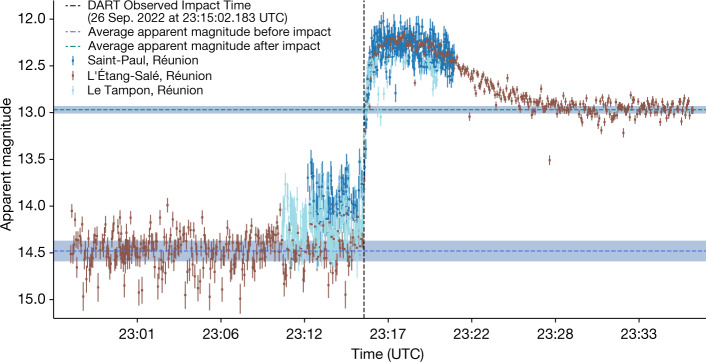
Apparent magnitude of Didymos before, during and after impact. The light curve of the Didymos binary system on 26 September 2022 during the DART spacecraft’s impact into Dimorphos as observed by three citizen astronomers located on Réunion Island using eVscopes. The dotted lines are the measured apparent magnitude before impact (bottom) and after the fast-moving ejecta dissipated after the impact (top). The shaded regions represent the standard deviation on the value of the dotted lines, and error bars represent sky background noise. Source data

Of the four eVscope data sets that included the moment of impact, three were suitable for photometric analysis because the observers recorded dark frames for image calibration. Thus, we conducted aperture photometry on these three data sets through a circular aperture radius of 13.6′′ or 750 km at the distance of Didymos The resulting apparent magnitudes measured over time are shown in Fig. 2.

Using data from the eVscopes located in L’Étang-Salé and Saint-Paul, Réunion, we calculate an impact time of UTC 23:15:02 ± 4 s on 26 September 2022, which agrees with the reported Earth-observed impact time of 23:15:02.183 UTC^1^, itself coming 38 s after the actual time of impact on the spacecraft due to light-travel time (private communication with J. Bellerose). Before the impact, we use the observations taken with the eVscope located in L’Étang-Salé, Réunion to measure an apparent visual magnitude, *m*_v_ = 14.48 ± 0.11 and a minimum magnitude (maximum brightness) *m*_v_ = 12.18 ± 0.03. As the fast-moving ejecta moved out of the photometric aperture, the magnitude increased to *m*_v_ = 12.96 ± 0.04. At a geocentric distance of 0.076 AU, a heliocentric distance of 1.05 AU and a phase angle of 53.28° at the time of impact, these correspond to absolute visual magnitudes of *H*_v_ = 18.12 ± 0.11, 15.83 ± 0.03 and 16.61 ± 0.04, respectively.

We estimate the effective mass in the fast-moving ejecta by calculating the change in effective cross-sections corresponding to magnitudes *H*_v_ = 15.83 ± 0.03 and 16.61 ± 0.04, and assuming an average density and albedo for Dimorphos. This is further detailed in the Mass of the ejecta section of the Methods. We must also assume an average particle radius, so we consider several scenarios.

To begin with, the particles must be small as evidenced by the long tail that developed in the antisolar direction several days after impact. We approximate an antisolar tail length of roughly 7 × 10^3^ km on the plane of the sky roughly 113.7 h after impact as shown in Fig. 1. Measurements from the 4.1-m Southern Astrophysical Research Telescope, at NSF NOIRLab’s Cerro Tololo Inter-American Observatory in Chile revealed the tail length to be more than 10^4^ km on the plane of the sky 2 days after impact^4^. Our measurement is shorter because of the smaller collecting area of the eVscope and its lesser sensitivity to low surface brightness. We measure a 3σ limiting magnitude of 17.01 ± 0.03 in this image stack consisting of 1,205 4-s exposures.

We then refer to active asteroid (596) Scheila, whose activity was probably caused by an impact, and whose average ejected dust size is predicted to be small on the basis of the observed effect of solar radiation sweeping^5^. From this, average dust radii spanning $\bar{a}$ of roughly 0.1–1.0 μm were estimated. We point out that larger dust sizes were also estimated for Scheila such as $\bar{a}$ ≅ 100 μm based on an approximate modelled particle range of *a* ≅ 1–10^4^ μm along a power-law distribution with an index *q* = 3.5 (refs. ^6,7^).

We first examine an average particle radius $\bar{a}$ ≅ 1 μm, as particles much smaller than this become less efficient at scattering visible light, and particles much larger than this are expected to persist longer in the photometric aperture. This particle radius then results in a mass of *m*_fe_ ≅ (7.0 ± 1.2) × 10^3^ kg contained in the fast-moving ejecta plume, respectively. We measured the speed of this fast-moving ejecta on the plane of the sky through the photometry aperture and find this fast-moving ejecta has a speed of *v*_fe_ ≅ 970 ± 50 m s^−^^1^. The resulting mass then corresponds to a kinetic energy of KE_fe_ ≅ (3.3 ± 0.6) × 10^9^ J. The relative kinetic energy of the DART spacecraft at the time of impact is ﻿${{\rm{K}}{\rm{E}}}_{{\rm{D}}}\,\cong \,(1.094\,\pm \,0.001)\times {10}^{10}$ J (ref. ^1^). This indicates that the observed fast-moving ejecta plume carried away roughly 30 ± 6% of the kinetic energy delivered by the DART spacecraft. This is comparable to impact simulations, which have shown that kinetic energy can be transferred from the impactor to the ejecta on the order of tens of percent^8–10^. Average particle sizes an order of magnitude larger than this would exceed the kinetic energy introduced by the DART spacecraft, so we do not further consider larger particles sizes as making up a substantial portion of the observed fast-moving ejecta plume. The lower end of the dust size estimations based on solar radiation pressure, $\bar{a}$ ≅ 0.1 μm, results in an estimated mass of *m*_fe_ ≅ (7.0 ± 1.2) × 10^2^ kg, corresponding to roughly 3.0 ± 0.6% of DART’s kinetic energy. These values probably represent underestimates on ejecta mass, given the assumed average dust radii, as only the particles with velocities high enough to escape Dimorphos (*ν* > *v*_e_ ≅ 0.087 ± 0.01 m s^−^^1^) will contribute to the measurable increase in cross-section^1,11,12^﻿.

**Fig. 3:**
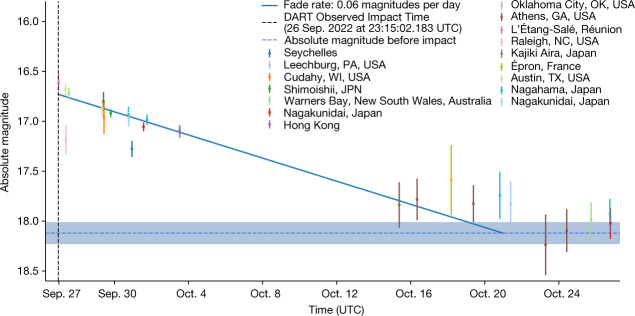
Fading of Didymos after impact. The absolute magnitude of the Didymos system faded over time after the brightening due to DART’s impact into Dimorphos. The solid blue line is a weighted, linear fit to magnitudes measured from just after impact on 26 September to 25 October 2022, after which measurements were consistent with the resting absolute magnitude. Some measurements between 15 and 25 October overlap the resting magnitude at the roughly 1*σ* level but others remain above it. Therefore, we consider the fading time may range between around 18 and 28 days after impact, with our best-fit model providing a fading time of 23.7 ± 0.7 days. The value of the resting absolute magnitude is calculated from the pre-impact average apparent magnitude plotted in Fig. 2. The error bars and shaded region represent the 1*σ* measurement uncertainties. Before 4 October 2022, there are two outlying observations that resulted in measurements that were too faint due to poor weather conditions, and we therefore do not include these points in the fitted line. Source data

To estimate the mass of ejecta contained within the coma, we measure Didymos’ fading rate. The absolute magnitudes of Didymos after impact are plotted in Fig. 3. An error-weighted linear fit indicates that it took roughly 23.7 ± 0.7 days for the dust to move out of the photometric aperture with velocity *v*_ce_ ≅ 0.37 ± 0.01 m s^−1^. We use an approximated impact relation (equation (6) in Methods)^13^ to relate this with the mass, density and speed of the DART impactor, and estimate the mass contained in the coma that resulted from the impact to be *m*_ce_ ≅ (1.3 ± 0.1) × 10^7^ kg. Considering the change in effective cross-section before impact and after the fast-moving ejecta dissipated, equation (3) in the Methods section results in an average dust radius of $\bar{a}$ ≅ 1.7 ± 0.3 mm.

We also estimate the particle radius in the observed coma by relating solar radiation pressure to the turn-around distance of the particles in the coma along the comet–Sun line, and the bulk velocity of the particles, *v*_ce_ (detailed in the Mass of the ejecta section of the Methods). It was found that particles reached distances﻿ *𝓁* of roughly 150–250 km in the solar direction before turning around^14^. We, then find average dust radii $\bar{a}$ roughly 2.8 ± 0.3–3.8 ± 0.4 mm in the coma, corresponding to a mass range of *m*_ce_ of roughly (1.3–2.2) × 10^7^ kg, in good agreement with our estimate above.

In all scenarios, we find that whereas the impact led to a significant increase in apparent magnitude, the overall mass loss in the observed fast-moving ejecta plum or slower-moving ejecta in the coma created by DART’s impact into Dimorphos was not totally disruptive.

**Fig. 4:**
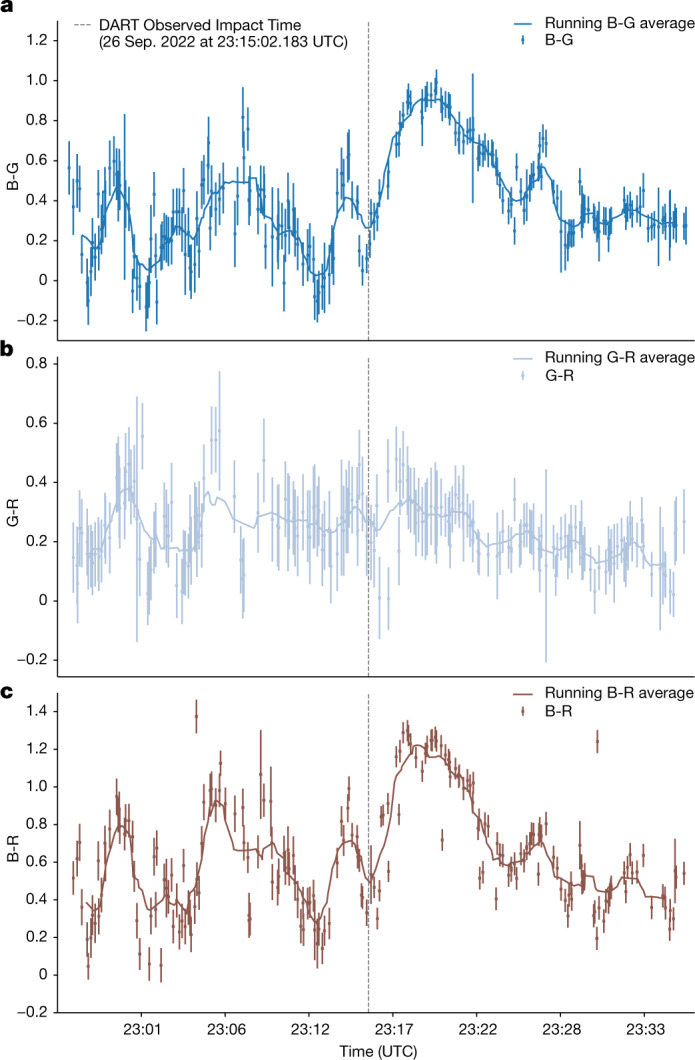
Colours of Didymos before, during and after impact. **a**–**c**, Measured B-G (**a**), G-R (**b**) and B-R (**c**) colours of Didymos over time as observed from L’Étang-Salé, Réunion. Error bars represent the noise introduced by the sky background magnitude. It is apparent in **a** and **c** that the Didymos binary system became redder directly after the DART spacecraft struck Dimorphos and that when the fast ejecta dissipated, the colours returned to their original colours. The G-R colours in **b** show no significant change in colour during the time of impact. Source data

We also measure the colour before, during and after DART’s impact into Dimorphos as seen in Fig. 4 to show a significant reddening at the time of impact. The colours appear to return to their original colour as the fast-moving ejecta plume dissipates. This initial reddening was also seen on 9P/Tempel 1 after the impact of NASA’s Deep Impact spacecraft and was determined to be caused by different sized particles having a range of velocities, causing the particle size distribution and the ejecta optical depth to change over time^15^.

If the reddened colour is interpreted as a proxy for material composition, we must consider this in the context of the colours of active and inactive small bodies in the Solar System. Typically, active bodies appear bluer in colour on average than their inactive counterparts, such as short-period comets versus Kuiper belt objects^16^. Some of these redder observed surface colours may be due to irradiation of organics^17^, which can efficiently mantle the surface of bodies such as the Kuiper belt objects^18,19^﻿. Furthermore, in general, the highest velocity ejecta from an impact is sourced from the material closest to the site of that impact^20^. The fast-moving ejecta plume may be more representative of the surficial material of Dimorphos than the material in the slower-moving coma. Whereas much of the fine-grain surface regolith on Dimorphos has probably been depleted through processes such as electrostatic removal^21^, it is possible that the remaining small particles have experienced some irradiation. Spectra indicate the presence heterogeneity of surface materials on Didymos, with various concentrations of probably hypersthene with a grain radius *a* < 25 μm, and olivine with a grain radius *a* < 45 μm (ref. ^22^). Laboratory simulations of space weathering showed that irradiation can cause significant reddening of olivine’s reflectance spectrum^23^. We emphasize that these posed interpretations are a few of possibly many.

## Methods

### Observations

The measurements in this work are derived from 23,240 images taken by 17 different eVscopes in the Unistellar network of eVscopes. At the time of this study, the Unistellar fleet of eVscopes contained three variations: the eVscope 1, eVscope eQuinox and the eVscope 2. All eVscopes have 112-mm diameter primary mirrors. The eVscope 1 and eQuinox both use a Sony IMX224LQR sensor and have a 37′ × 28′ field of view and pixel scale of 1.72′′ per pix, whereas the eVscope 2 uses a Sony IMX347LQR sensor and has a 45′ × 34′ field of view and pixel scale of 1.33′′ per pix. The detectors have RGB photosensors arranged as Bayer filter matrices. Exposure times of individual images varied between observers from 1.0 to 4.0 s but remained constant throughout each individual data set. Images were taken consecutively over ranges of 10–80 min. In this work, we report observations taken from 26 September to 31 October 2022 UT. Over this time range, the observing parameters of Didymos ranged from geocentric distances of 0.076 to 0.109 AU, heliocentric distances of 1.046 to 1.018 AU and phase angles of 53.2° to 73.8°. Figure 1 shows two of the tails that formed after impact as seen on 1 October 2022, when the position angle was 60.4° in the projected antisolar direction and 27.6° in the projected anti-velocity direction. In each data set, the limiting magnitude is between 15 and 16 mag, except for the stacked image that shows the tails in Fig. 1, which consists of 1,250 4-s images. For this data set, we measure a 3σ limiting magnitude of 17.01 ± 0.03. We further summarize the observation circumstances in Extended Data Table 1.

### Aperture photometry

To measure the magnitude of Didymos in the images, we first reduce the data submitted by Unistellar citizen astronomers. Unistellar citizen astronomers submit science images with their chosen parameters (as listed above) as well as dark frames for calibration that are recorded immediately after the science observations. The dark frames are median combined and subtracted from the science images. The Unistellar network is not prompted at this point to take flat frames to further calibrate the data. In general, we find the errors on photometric measurements are dominated by photon noise and sky rather than the lack of a flat-field correction. We also aim to keep Didymos in the middle of the detector when conducting observations to further mitigate the lack of a flat frame calibration. Before conducting aperture photometry, the science images are aligned and median combined in time; however, the observations conducted during the DART impact shown in Fig. 2 were measured from images that were not stacked because of the speed of brightening on impact. The photometry measurements on the observations conducted after the impact were conducted on median-combined images that vary from 17 to 30 images. The measurements are then averaged and plotted at the midpoint in time of the conducted observation. We choose an aperture radius of 13.6′′ corresponding to 75 km at the distance of Didymos through which we measure the flux. The point spread function (PSF) of Didymos after the impact depends on the data set. Extended Data Table 1 lists the average full-width at half-maximum of the gaussian-fitted PSFs of the stars in each data set. The most egregious cases were due to out of focus eVscopes. We find the full-width at half-maximum of the PSF of Didymos is roughly 9.0 ± 1.0% larger than that of the stellar PSFs. Our chosen aperture is large enough to measure all the flux from the Didymos system throughout all the submitted data sets. We subtract the flux of the sky background measured through an annulus centred on the target that ranges from 22.21′′ to 30.75′′ in radius, corresponding to roughly 1,218 to 1,686 km at the distance of Didymos. We use stars in the field with known Gaia magnitudes to convert our instrumental flux values to apparent magnitudes, *m*_V_. These magnitudes are plotted in Fig. 2. When measuring the magnitude before impact and after the fast-moving ejecta dissipated, we use observations from the eVscope in L’Étang-Salé, Réunion on 26 September 2022. This eVscope was able to reach the greatest sensitivities as it was contained in a protective dome that blocked wind and stray light.

To obtain the colours of the Didymos binary system, the colour channels of the detectors must be isolated. In a Bayer filter matrix of blue (B), green (G) and red (R) photosensors, the first 2 × 2 section of pixels is arranged as [R, G; G, B]. This pattern repeats to fill the size of the detector. We isolate each colour channel on the basis of this known pattern of the photosensors and conduct aperture photometry as described above. We calibrate the instrumental fluxes using calibrator stars with known Gaia magnitudes in the background of each image. We measure the aperture fluxes of those calibrator stars in each colour channel and, combined with their known Gaia magnitudes, use them to convert the Didymos aperture fluxes in those channels to B, G and R magnitudes^24^. We plot the resulting B-G, G-R and B-R colours, the B-G and B-R colours in Fig. 4.

### Mass of the ejecta

Apparent magnitudes *m*_V_ are converted to absolute magnitudes, *H*_V_, which represents the magnitude of the object with heliocentric and geocentric distance (*r*_H_ and ∆, respectively) of 1 AU and at phase angle, *α* = 0°. The correction is

1 $${H}_{{\rm{V}}}={m}_{{\rm{V}}}-5{\log }_{10}\left({r}_{{\rm{H}}}\Delta \right)-\beta \alpha $$

where *βα* is the phase function that represents the dependence on sunlight scattering by the dust particle at an angle *α* in degrees. We assume a linear phase function with phase coefficient, *β* = 0.035, a typical value for S-type asteroids such as Didymos^25–29^﻿. With this, our measured absolute magnitude before the impact of *H*_V_ = 18.12 ± 0.11 agrees with past measurements of *H*_V_ = 18.16 ± 0.04 of the binary system^30^. Absolute magnitudes are plotted in Fig. 3. Next, we estimate the effective cross-sections with

2 $${C}_{{\rm{e}}}=\frac{\pi \left(2.25\times {10}^{16}\right)}{{p}_{{\rm{v}}}}{10}^{-0.4}\left[{H}_{{\rm{V}}}-{V}_{\odot }\right]$$

where *p*_v_ ≅ 0.15 ± 0.02 is the albedo of the Didymos system measured by the DRACO camera^1^, *H*_V_ is the absolute magnitude we measure for the Didymos system and *V*_⊙_ ≅ −26.77 is the apparent magnitude of the Sun. For Didymos, we find an effective scattering cross-section *C*_e_ ≅ 0.53 ± 0.06 km^2^ before impact, *C*_e_ ≅ 4.35 ± 0.13 km^2^ at Didymos’ peak in brightness just after impact and *C*_e_ ≅ 2.17 ± 0.09 km^2^ after the fast-moving ejecta moved out of the photometric aperture. A cross-section *C*_e_ ≅ 0.53 ± 0.06 km^2^ indicates an effective radius *r*_e_ ≅ 411 ± 22 m, which is consistent with previous radar measurements of Didymos finding a volume equivalent radius of *r*_e_ ≅ 390 ± 15 m (ref. ^11^). The mass is related to the effective cross-section ﻿by

3 $${m}_{{\rm{e}}}=\frac{4}{3}\rho \bar{a}{C}_{{\rm{e}}}$$

where *ρ* ≅ 2,400 ± 250 kg m^−3^ is the bulk density of the Didymos system^1^, and we adopt $\bar{a}=\sqrt{{a}_{\min }{a}_{\max }}$ for the mean dust radius among particles having a size range ${a}_{\min }\le a\le {a}_{\max }$. We examine particles with mean radii $\bar{a}$ ≅ 0.1–1 μm in the fast-moving ejecta as was found for the ejecta in the impacted asteroid Scheila^5^. The change in effective cross-section measured at peak brightness and at the levelled brightness after the fast-moving ejecta dissipated then allows us to measure the change in mass, or the mass contained in the fast-moving ejecta plume with equation (3). We do the same for the mass lost in the coma of slower-moving particles by examining the change in cross-section from before impact and after impact, when the fast-moving ejecta dissipated from the photometric aperture. However, instead of assuming a dust size within the coma, we estimate the dust size in two ways. First, we estimate the mass in the coma on the basis of the fading time of Didymos after impact and the speed of the particles through a process explained in the following section. Equation (3) then gives an estimate on the average dust radius. We also estimate the dust radius by connecting the distance a particle can reach in the Solar direction before being turned around by Solar radiation pressure to its initial speed using

4 $$B=\frac{{u}^{2}{{r}_{{\rm{H}}}}^{2}}{2G{M}_{\odot }{\mathcal{l}}}$$

where *B* is a dimensionless radiation pressure factor, *G* = 6.67 × 10^−11^ m^3^ kg^−^^1^ s^−^^2^ is the gravitation constant, *M* is the mass of the Sun, *r*_H_ ≅ 1.04 AU is the heliocentric distance of Dimorphos on 1 October 2022, when the turn-around distance 𝓁 was measured to reach roughly 150–200 km (ref. ^14^) and *u* is the initial velocity of the particles that we measure as *u* ≅ *v*_ce_ ≅ 0.37 ± 0.01 m s^−^^1^ from the fading rate of Didymos. *B* is the ratio of acceleration due to Solar radiation pressure to the acceleration due to Solar gravity expressed as

5 $$B=\frac{{KQ}_{{\rm{pr}}}}{\rho a}$$

where *K* = 5.7 × 10^-4^ kg m^−2^ is a constant, *Q*_pr_ is the radiation pressure coefficient often assume to be 1, *ρ* is the particle density and we examine the average dust *a* = $\bar{a}$. Again, we examine dust densities equivalent to the bulk density of the Didymos system^1^. Assuming *ρ* ≅ 2,400 ± 250 kg m^−3^ gives *B* ≅ (0.24 ± 0.02)/$\bar{a}$ μm. We point out that using a﻿﻿  density *ρ* ≅ 3,480 ± 80 kg m^−^^3^, which is the average density of LL ordinary chondrite material^31^ as associated with S-type asteroid Didymos^32^, also results in millimetre-sized particles corresponding to masses within the range we estimate when assuming *ρ* ≅ 2,400 ± 250 kg m^−3^.

### Speed and energy of the ejected dust

To estimate the energy carried by the mass in that fast-moving ejecta plume and slower-moving coma, we obtain their speeds. The fast-moving ejecta can be measured visually, following the plume on the detector over time. Furthermore, we measure the crossing time, *t*_c,_ of the particles in the photometric aperture. This is the time between the moment of impact and the moment the magnitude settled to *m*_v_ = 12.96 ± 0.04. We determine the peak time and dissipation time of the fast-moving ejecta by analysing the magnitudes binned into rolling bins of five images to determine peak time and 15 images for the settling time. We then choose times when the residuals were within the respective measured errors on the peak magnitude and magnitude after fast-moving ejecta dissipation. We find a crossing time *t*_c_ ≅ 775 ± 40 s over a photometric aperture radius of 13.67′′, which is equivalent to roughly 750 km at the distance of Didymos. Therefore, we obtain a speed of *v* ≅ 970 ± 50 m s^−1^.

In the slower-moving ejecta that makes up the coma, we estimate the particle speed from the fading time of Didymos after impact. This is the time between the moment of impact and the moment the magnitude increased back to Didymos’ original absolute magnitude, *H*_V_ = 18.12 ± 0.11. Then, assuming equal projectile and target densities, we make a simple estimation of the mass of the ejecta *m*_e_ by the impact relation

6 $${m}_{{\rm{e}}}=A\times {{M}_{{\rm{P}}}\left(\frac{u}{{U}_{{\rm{P}}}}\right)}^{s}$$

where *U*_P_ ≅ 6,144.9 ± 0.3 is the impact speed of the DART spacecraft^1^, *M*_P_ ≅ 579.4 ± 0.7 is the mass of the DART spacecraft on impact^1^, *u* ≅ *v*_ce_ ≅ 0.37 ± 0.01 m s^−1^ is the bulk velocity of the particles in the ejecta, and for consistency with past works *A* = 0.01 is a constant and *s* is an index that that we approximate to *s* = −1.5 but depends on the material^7,13^. The density of Dimorphos is assumed to be the same as the bulk density of the Didymos system in the calculations, however, we emphasize that the density of Dimorphos alone is not measured. The density of the main body of the DART spacecraft (without the solar panels) was roughly 270 kg m^−3^ at impact time^33^. For the sake of this simple approximation, we take the densities of the spacecraft and Dimorphos to be similar enough, because we expect lower densities with decreasing diameter for S-type asteroids^34^ and assume that the spacecraft was stopped by Dimorphos rather than flying straight through it. We also emphasize that this approximation comes with the caveat that impact physics on small asteroids are still not well-understood, so this impact relation serves as a rough estimate^34^.

With the estimated masses, *m* and speeds, *v* of the ejecta, we can estimate the kinetic energy,﻿﻿ KE carried by the initial fast-moving ejecta as well as the coma by﻿ ${\rm{K}}{\rm{E}}=(1/2)m{v}^{2}$. As a tool to choose appropriate dust size test-cases, we compared the estimated kinetic energies to the kinetic energy introduced by the DART spacecraft where mass on impact is *M*_P_ ≅ 579.4 ± 0.7 kg and velocity on impact is *v* = *U*_P_ ≅ 6,144.9 ± 0.3 m s^−1^. We also compare this to the orbital energy, *E*_O_ of Dimorphos around Didymos before and after the orbital period change of −33.0 ± 1.0 min (ref. ^35^) using

7 $${E}_{{\rm{O}}}=\frac{-G{M}_{{\rm{Did}}}{m}_{{\rm{Dim}}}}{2r}$$

where ﻿${M}_{{\rm{D}}{\rm{i}}{\rm{d}}}\,\cong \,(5.6\,\pm \,0.5)\times {10}^{11}$ kg, ${m}_{{\rm{D}}{\rm{i}}{\rm{m}}}\,\cong \,4.3\times {10}^{9}$ kg and *r* is the semimajor axis of the orbit of Dimorphos around Didymos^1,36^. Before the impact, the semimajor axis was measured to be *r* = 1.206 ± 0.035 km^1^. We can estimate the original orbital energy of Dimorphos to be *E*_O_ ≅ −(6.6 ± 0.6) × 10^7^ J. With the orbital period decrease, Kepler’s third law gives a new semimajor axis of *r* ≅ 1.2 ± 0.1 km resulting in a change of orbital energy of ﻿$\Delta {E}_{{\rm{O}}}\cong \,(2.1\pm \,0.6)\times {10}^{6}\,{\rm{J}}$. These simple approximations are sufficient for the scope of this work, which aims to use these estimates as a means of providing a check on the reasonability of the estimated masses of the ejecta.

## Online content

Any methods, additional references, Nature Portfolio reporting summaries, source data, extended data, supplementary information, acknowledgements, peer review information; details of author contributions and competing interests; and statements of data and code availability are available at 10.1038/s41586-023-05852-9.

### Source data

Source Data Fig. 2

Source Data Fig. 3

Source Data Fig. 4

Source Data Extended Data Table 1

## Figures and Tables

**Extended Data Table 1:**
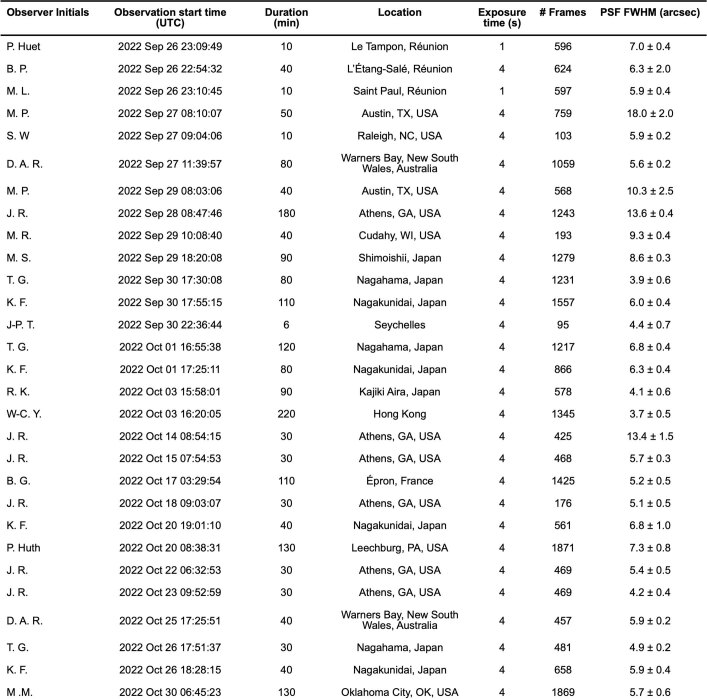
Summary of observations

Listed are the observations used in the above analyses from 2022 Sep 2022 to 30 Oct 2022. Observations were conducted by citizen astronomers across the World. The FWHM of the point spread function (PSF) of the stars is representative of the average “seeing” measured in each set of observations.

Source data

## Data Availability

The Unistellar network of citizen astronomers have the option to upload their FITS (Flexible Image Transport System) images to an Amazon Web Services server rented by the Unistellar Corporation. This data are then available upon request. The resulting photometry used in the data analyses are made available on the corresponding author’s public GitHub repository in the form of CSV files corresponding to the figures and the Extended Data Table. The repository also contains the FITS images used in Fig. 1. The data are located in the corresponding author’s public GitHub repository (https://github.com/Ariel-Graykowski/DART_Unistellar)^37^. Source data are provided with this paper.

## Code availability

The SETI/Unistellar pipeline used to dark-subtract and stack the astronomical images and conduct aperture photometry is currently located on Unistellar’s private GitHub. These codes are available on request. Python codes used to create the figures and conduct the main data analysis are located in the corresponding author’s public GitHub repository (https://github.com/Ariel-Graykowski/DART_Unistellar)^37^. Source data are provided with this paper.
